# Time to think beyond door to balloon time: significance of total ischemic time in STEMI

**DOI:** 10.1186/s43044-021-00221-1

**Published:** 2021-10-29

**Authors:** Sanam Khowaja, Salik Ahmed, Rajesh Kumar, Jehangir Ali Shah, Kamran Ahmed Khan, Naveed Ullah Khan, Tahir Saghir, Syed Nadeem Hasan Rizvi, Nadeem Qamar, Musa Karim

**Affiliations:** National Institute of Cardiovascular Diseases (NICVD), Karachi, Pakistan

**Keywords:** ST-segment elevation myocardial infarction, Total ischemic time, Door to balloon, Primary percutaneous coronary intervention, In-hospital outcomes

## Abstract

**Background:**

Significance of total ischemic time (TIT) in the context of ST-segment elevation myocardial infarction (STEMI) is still controversial. Therefore, in this study, we have evaluate the association of TIT with immediate outcomes in STEMI patients in whom recommended door to balloon (DTB) time of less than 90 min was achieved.

**Results:**

A total of 5730 patients were included in this study, out of which 80.9% were male and median age was 55 [61–48] years. The median DTB was observed to be 60 [75–45] min and onset of chest pain to emergency room (ER) arrival time was 180 [300–120] min. Prolonged TIT was associated with poor pre-procedure thrombolysis in myocardial infarction (TIMI) flow grade (*p* = 0.022), number of diseased vessels (*p* = 0.002), use of intra-aortic balloon pump (*p* = 0.003), and in-hospital mortality (*p* = 0.002). Mortality rate was 4.5%, 5.7%, and 7.8% for the patients with TIT of ≤ 120 min, 121 to 240 min, and > 240 min, respectively. Thirty days’ risk of mortality on TIMI score was 4.97 ± 7.09%, 5.01 ± 6.99%, and 7.12 ± 8.64% for the patients with TIT of ≤ 120 min, 121 to 240 min, and > 240 min, respectively.

**Conclusions:**

Prolonged total ischemic was associated with higher in-hospital mortality. Therefore, TIT can also be considered in the matrix of focus, along with DTB time and other clinical determinants to improve the survival from STEMI.

## Background

Both American College of Cardiology (ACC)/American Heart Association (AHA)/Society for Cardiovascular Angiography and Interventions (SCAI) and European Society of Cardiology (ESC) guidelines recommend primary percutaneous coronary intervention (PCI) for early reperfusion of the ST-segment elevation myocardial infarction (STEMI) for patients presenting to emergency room (ER) within 12 h of onset of chest pain [1, 2]. The reperfusion therapy recovers maximum myocardial damage if performed within initial few hours of chest pain and with every passing hour the recovery decreases sharply [3]. Percutaneous intervention has received preference over pharmacologic therapy for reperfusion of STEMI patients due to its ability to rapidly restore the patency of affected artery [4]. Hence, the ER arrival to the ballooning time, known as door to balloon (DTB) time, gained immense importance among the cardiologist and is considered to be the most important quality indicator for primary PCI [5]. Emphasis on reduction of DTB time was made in the management guidelines of STEMI and optimal DTB time of ≤ 90 min is recommended [1, 2, 6].

However, less attention was given to the other delays in the timeline of STEMI such as time between onset of chest and PCI capable hospital arrival [5]. The total ischemic time (TIT), time between onset of chest and ballooning or device activation, can be a more strong prognostic marker than DTB time as increase in microvascular obstruction area is reported to be associated with prolonged ischemia [7]. TIT was reported to be an independent predictor of infarct size and short and long term mortality in patients with STEMI [4, 5, 8, 9]. The optimal prognostic cutoff for TIT is debatable. It was reported that the reduction in DTB time has no added advantage in the late presented STEMI patients [7, 10]; therefore, the efforts of DTB time reduction failed to decrease the mortality rate of STEMI patients [11, 12]. Various factors have been reported to play role in prolongation of TIT such as unawareness and patients failure to recognize cardiac symptoms, unavailability of attendant, transportation, finance, and nearby facility [9].

Consequently, it is well established that DTB time is very important in identifying in-hospital outcomes and long term morbidity and mortality in the STEMI; however, significance of total ischemic time in STEMI patients is still debatable. Moreover, there is paucity of local data which further enlightened its significance for further research. Therefore, the aim of this study was to evaluate the association of TIT with immediate outcomes in STEMI patients in whom recommended DTB time of less than 90 min was achieved.

## Methods

### Study population

This study was conducted on selected patients from a prospectively collected institutional data registry. A single center hospital-based registry was established in January 2015 at the National Institute of Cardiovascular Diseases (NICVD), Karachi, Pakistan. Prior to inclusion, informed consent was taken explaining purpose and benefits of the study. Collected data were accessible to authorized personnel only and confidentiality was maintained at all stage of extraction of data and results. NICVD is one of the largest public sector tertiary care cardiac hospital and training center in Pakistan where primary PCI is being offered as the standard treatment 24/7 since 2010. All the procedures were performed as per the ACC/AHA guidelines by consultant cardiologist with experience of more than 5 years. Any contraindication to dual antiplatelet therapy was ruled out by taking brief history of the patient and an echocardiographic screening was done to assess any potential mechanical complications.

### Study variables

Study was commenced after approval of the institutional ethical review committee. In this study, we included patients presented within 12 h of onset of chest pain, diagnosed with STEMI and underwent primary PCI during 1st June 2015 to 28th February 2018. Patients with prior history of any cardiac related surgery or intervention and patients with DTB time of > 90 min, due to any reason, were also excluded from the study. The TIT was measured on hospital arrival and was defined as the duration (in minutes) between onset of chest pain and ER arrival plus the duration (in minutes) between ER arrival and device activation. The DTB time was defined as the duration (in minutes) between ER arrival and device activation. Variables extracted from the registry were patient’s demography, risk profile, intra procedural characteristics, post procedural components, presenting KILLIP class, time variables, and in-hospital mortality.

### Statistical analysis

Patients were grouped into three based on TIT: Group 1, TIT ≤ 120 min; Group 2, TIT between 121 to 240 min; and Group 3, TIT > 240 min. All the variables were entered into the IBM SPSS Statistics for Windows, Version 21.0. (IBM Corp., Armonk, NY, US) for data analysis. Frequency and percentages were calculated for all the categorical variables. Continuous variables were assessed for normality of distribution by applying Kolmogorov–Smirnov normality test and appropriate summary measure such as mean ± standard deviation (SD) or median [interquartile range (IQR)] were computed. Records with missing information regarding disease anatomy, in-hospital outcome, and other study variables were excluded from the analysis. The receiver operating characteristic (ROC) curve analysis was performed and area under the curve (AUC), optimal prognostic cutoff value of TIT and its sensitivity and specificity were calculated. Chi-square test was performed to assess the differences in various characteristics and outcomes by different TIT groups. Multivariable logistic regression analysis was performed to assess the predictors of mortality taken demographic characteristics (such as gender and age), co-morbid conditions (such as diabetes, hypertension, smoking, and positive family history of coronary artery diseases), and TIT as explanatory variables based on results of univariate comparison and clinical judgment and odds ratio (OR) and 95% confidence interval (CI) were reported. Throughout the analysis, *p* ≤ 0.05 was the criteria for statistical significance.

## Results

A total of 5730 patients were selected, of them 4637 (80.9%) were males and median age was 55 (61–48) years. The median of DTB was observed to be 60 (75–45) min and chest pain ER arrival time was 180 (300–120) min. A total of 2978 (52%) patients were hypertensive, 1712 (29.9%) diabetic, 1636 (28.6%) smokers, and 263 (4.6%) had family history of CAD. Prolonged TIT was found to be associated with age (*p* < 0.001), gender (*p* < 0.001), diabetes (*p* = 0.039), and hypertension (*p* = 0.021). Baseline clinical and demographic characteristics stratified by the total ischemic time are presented in Table 1.

**Table 1:** Baseline clinical and demographic characteristics stratified by the total ischemic time

Characteristics	Total	Total ischemic time (min)			*p* value
		Up to 120	121 to 240	More than 240	
Total	5730	418 (7.3%)	2199 (38.4%)	3113 (54.3%)	–
Age (years)					
Median [IQR]	55 [61–48]	52.5 [60–45]	55 [60–47]	55 [62–48]	< 0.001*
Up to 40 years	608 (10.6%)	53 (12.7%)	234 (10.6%)	321 (10.3%)	0.025*
41 to 50 years	1675 (29.2%)	139 (33.3%)	657 (29.9%)	879 (28.2%)	
51 to 60 years	2004 (35%)	147 (35.2%)	767 (34.9%)	1090 (35%)	
> 60 years	1443 (25.2%)	79 (18.9%)	541 (24.6%)	823 (26.4%)	
Gender					
Male	4637 (80.9%)	369 (88.3%)	1832 (83.3%)	2436 (78.3%)	< 0.001*
Female	1093 (19.1%)	49 (11.7%)	367 (16.7%)	677 (21.7%)	
Medical history					
Diabetes	1712 (29.9%)	123 (29.4%)	616 (28%)	973 (31.3%)	0.039*
Hypertension	2978 (52%)	195 (46.7%)	1123 (51.1%)	1660 (53.3%)	0.021*
Smoke	1636 (28.6%)	122 (29.2%)	647 (29.4%)	867 (27.9%)	0.438
Family history	263 (4.6%)	20 (4.8%)	89 (4%)	154 (4.9%)	0.298
KILLIP class					
I	5070 (88.5%)	376 (90%)	1977 (89.9%)	2717 (87.3%)	0.009*
II	287 (5%)	16 (3.8%)	111 (5%)	160 (5.1%)	
III	159 (2.8%)	9 (2.2%)	43 (2%)	107 (3.4%)	
IV	214 (3.7%)	17 (4.1%)	68 (3.1%)	129 (4.1%)	
Symptom onset to hospital arrival time (min)					
Median [IQR]	180 [300–120]	60 [60–30]	120 [160–120]	283 [385–240]	< 0.001*
Door to balloon time (min)					
Median [IQR]	60 [75–45]	45 [55–37]	55 [71–45]	65 [75–49]	< 0.001*
Total ischemic time (min)					
Median [IQR]	255 [355–180]	101 [110–85]	185 [213–165]	341 [446–285]	< 0.001*

*IQR* interquartile range

*Significant at 5%

Angiographic and procedural characteristics stratified by the TIT are presented in Table 2. Prolonged TIT was associated with pre-procedure TIMI flow (*p* = 0.022), number of diseased vessels (*p* = 0.002), use of IABP (*p* = 0.003), and side branch involvement (*p* = 0.008).

**Table 2:** Angiographic and procedural characteristics stratified by the total ischemic time

Characteristics	Total	Total ischemic time (min)			*p* value
		Up to 120	121 to 240	More than 240	
Total	5730	418 (7.3%)	2199 (38.4%)	3113 (54.3%)	–
Pre-procedural TIMI flow					
No flow	3272 (57.1%)	240 (57.4%)	1244 (56.6%)	1788 (57.4%)	0.022*
I	1013 (17.7%)	70 (16.7%)	402 (18.3%)	541 (17.4%)	
II	863 (15.1%)	66 (15.8%)	297 (13.5%)	500 (16.1%)	
III	582 (10.2%)	42 (10%)	256 (11.6%)	284 (9.1%)	
Number of vessels involved					
None	14 (0.2%)	0 (0%)	7 (0.3%)	7 (0.2%)	0.002*
Single vessel (SVD)	2278 (39.8%)	177 (42.3%)	938 (42.7%)	1163 (37.4%)	
Multi vessels (MVD)	3430 (59.9%)	240 (57.4%)	1249 (56.8%)	1941 (62.4%)	
Left main (LM)	8 (0.1%)	1 (0.2%)	5 (0.2%)	2 (0.1%)	
Infarct related artery					
None	15 (0.3%)	0 (0%)	7 (0.3%)	8 (0.3%)	0.884
LAD	3097 (54%)	242 (57.9%)	1184 (53.8%)	1671 (53.7%)	
Right coronary artery	1828 (31.9%)	121 (28.9%)	712 (32.4%)	995 (32%)	
Left circumflex	660 (11.5%)	48 (11.5%)	244 (11.1%)	368 (11.8%)	
Ramus	27 (0.5%)	2 (0.5%)	10 (0.5%)	15 (0.5%)	
Left main	26 (0.5%)	3 (0.7%)	10 (0.5%)	13 (0.4%)	
Diagonal	64 (1.1%)	2 (0.5%)	27 (1.2%)	35 (1.1%)	
Others	13 (0.2%)	0 (0%)	5 (0.2%)	8 (0.3%)	
Side branch involvement					
Yes	645 (11.3%)	65 (15.6%)	227 (10.3%)	353 (11.3%)	0.008*
No	5085 (88.7%)	353 (84.4%)	1972 (89.7%)	2760 (88.7%)	
Left ventricular dysfunction					
Yes	3239 (56.5%)	232 (55.5%)	1210 (55%)	1797 (57.7%)	0.134
No	2491 (43.5%)	186 (44.5%)	989 (45%)	1316 (42.3%)	
Thrombus grading					
No	409 (7.1%)	28 (6.7%)	166 (7.5%)	215 (6.9%)	0.346
Possible	591 (10.3%)	43 (10.3%)	217 (9.9%)	331 (10.6%)	
Small	552 (9.6%)	35 (8.4%)	220 (10%)	297 (9.5%)	
Moderate	883 (15.4%)	60 (14.4%)	345 (15.7%)	478 (15.4%)	
Large	746 (13%)	53 (12.7%)	315 (14.3%)	378 (12.1%)	
Total	2549 (44.5%)	199 (47.6%)	936 (42.6%)	1414 (45.4%)	
Intra-aortic balloon pump (IABP) used					
Yes	193 (3.4%)	10 (2.4%)	55 (2.5%)	128 (4.1%)	0.003*
No	5537 (96.6%)	408 (97.6%)	2144 (97.5%)	2985 (95.9%)	
Export catheter					
Yes	1887 (32.9%)	148 (35.4%)	736 (33.5%)	1003 (32.2%)	0.339
No	3843 (67.1%)	270 (64.6%)	1463 (66.5%)	2110 (67.8%)	

*LAD* left anterior descending artery, *TIMI* thrombolysis in myocardial infarction

*Significant at 5%

**Fig. 1:**
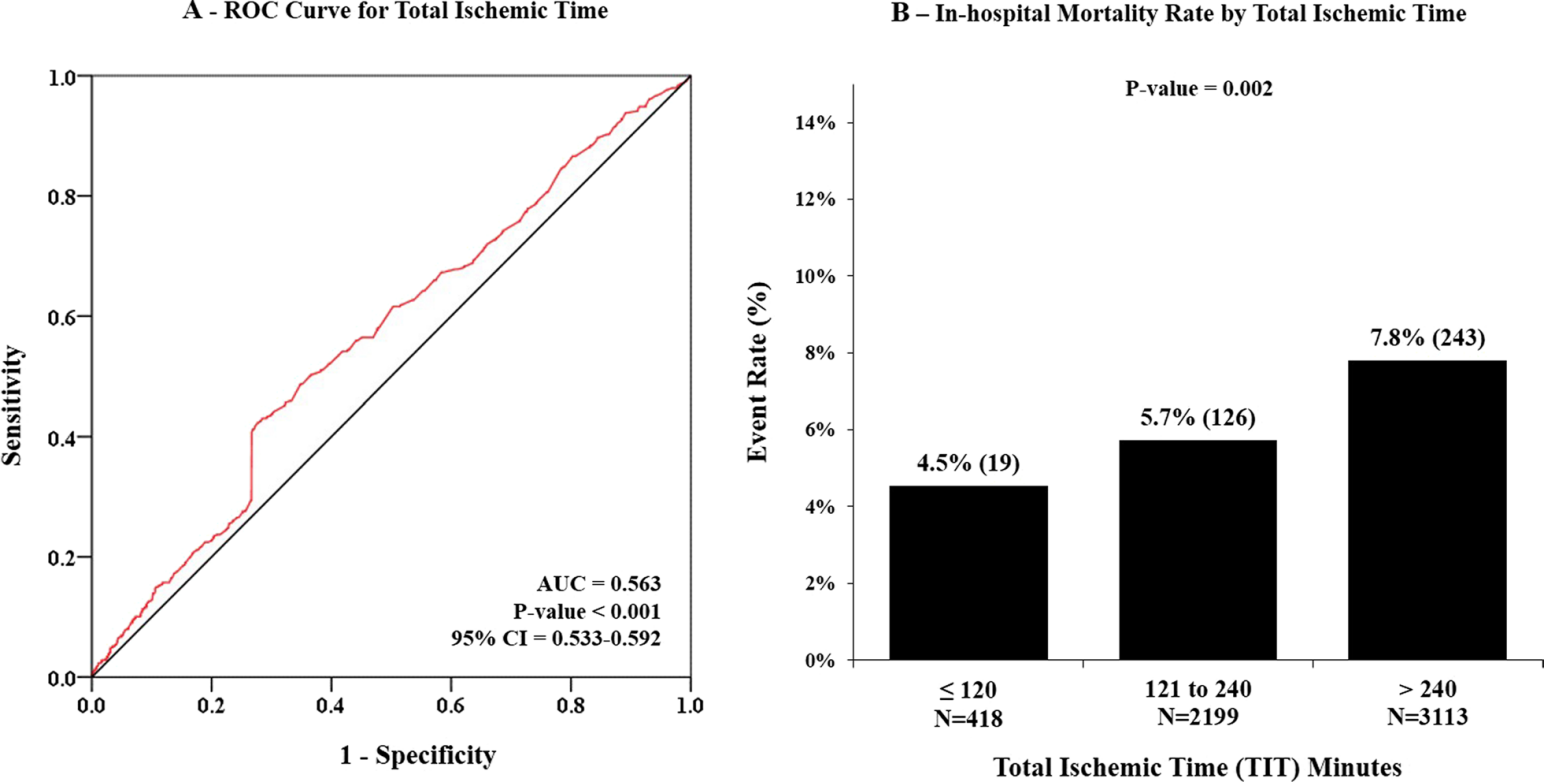
**A** Receiver operating characteristic (ROC) curve and **B** in-hospital mortality rate stratified by the total ischemic time

Post-procedure TIMI flow grade 0 was observed in 94 (1.6%), I in 47 (0.8%), II in 228 (4.0%), and III in 5361 (93.6%) patients. In-hospital mortality rate was 388 (6.8%) and mortality rate was 4.5%, 5.7%, and 7.8% for the patients with TIT of ≤ 120 min, 121 to 240 min, and > 240 min, respectively. Thirty days risk of mortality on TIMI score was 2.2% (4.4–1.6%), 2.2% [4.4–1.6%], and 4.4% [7.3–2.2%] for the patients with TIT of ≤ 120 min, 121 to 240 min, and > 240 min, respectively. The optimal cutoff value of TIT based on ROC curve analysis was found to be 297 min (rounded of to 300 min), with sensitivity of 50.26% and specificity of 63.33%. ROC curve and in-hospital mortality rate stratified by the TIT are presented in Fig. 1.

Multivariable logistic regression analysis for in-hospital mortality is presented in Table 3. On multivariable logistic regression, female gender (OR = 1.29, *p* = 0.046), age (OR = 1.04, *p* < 0.001), diabetes (OR = 1.91, *p* < 0.001), and TIT (OR = 1.05, *p* < 0.001) were significant predictors of in-hospital mortality.

**Table 3:** Multivariable logistic regression analysis for in-hospital mortality

Predictors	Odds ratio (OR)	95% CI for OR	*p* value
Age (years)	1.04	1.03–1.05	< 0.001*
Female gender	1.29	1–1.65	0.046*
Diabetic	1.91	1.54–2.37	< 0.001*
Hypertensive	1.08	0.87–1.34	0.499
Smoker	0.89	0.68–1.16	0.376
Positive family history	1.15	0.7–1.89	0.588
Total ischemic time (h)	1.05	1.02–1.07	< 0.001*

Dependent variable in-hospital mortality [yes]

Hosmer and Lemeshow Test (chi-square = 5.876, *df* = 8, *p* = 0.661)

*OR* odds ratio, *CI* confidence level

*Significant at 5% level of significance

## Discussion

In this study, our aim was to assess the effect of TIT on mortality of STEMI patients in whom the recommended DTB time of < 90 min was achieved. We found that TIT is a good prognostic marker, mortality rate is linearly associated with TIT and every delay of an hour after onset of chest pain increases risk of mortality nearly by 5% (OR = 1.05). The optimal prognostic cutoff value for the TIT was 300 min, after which the risk of mortality increases exponentially. In our setup, more than half (54.3%) of the STEMI patients had TIT of more than 4 h. Prolonged TIT was observed to be associated with age and female gender, and patients with prolonged TIT had low TIMI flow grades, multivessel involvement, and increased use of IABP.

The prognostic importance of TIT is well documented by various studies in the past. De Luca et al. [8] reported shorter TIT in patients with successful reperfusion and every half an hour delay was reported to be associated with an increased relative risk of mortality at 1 year. Khalid et al. [9], in an editorial, highlighted that the myocardium gets injured with every passing second; therefore, TIT should be targeted in STEMI patients to reduce the mortality rate. Prolonged TIT is a problem not specific to a certain geography or population, it exists across the world with varying degrees of intensity. For example, a study conducted in Australian population by Chandrasekhar et al. [13] reported prolonged TIT (> 240 min) for more than one third (34.2%) of the STEMI patients. They also reported that TIT was strongly correlated and found as an independent predictor of major adverse cardiovascular events (MACE). An Indian study by Doddipalli et al. [14] reported that lack of awareness and time taken by patients in recognizing symptom were the main contributors to prolonged TIT and the mean TIT was reported to be significantly higher among expired patients, (8.0 ± 3.6 h vs. 6.2 ± 2.8 h; *p* < 0.05) as compared to alive patients. Results from a Korean nationwide registry by Kim et al. [15] observed that shorter (< 3 h) TIT was associated with reduced risk of mortality at one month. According to Koifman et al. [16], utilization of mobile coronary care unit (CCU) is an effective strategy to reduce TIT which subsequently reduced 1-year mortality among STEMI patients. Similarly, Solhpour et al. [5] conducted a study at a STEMI center in US revealed TIT as a better predictor of mortality than DTB time. They documented that TIT was more than 4 h for nearly one-third of the STEMI patients and reported to be a good predictor of infarct size and 30-day mortality than DTB time.

Hence, most of the studies addressing the impact of TIT in STEMI management, including our study, agreed to the fact that TIT holds prognostic utility. In fact, in a number of cases, it proved to be a better predictor of mortality (immediate, short, and long term) than DTB time [5, 8, 9, 13–16]. One of underlying mechanism of increased mortality with prolongation of ischemic time, as reported in animal model study, is that infarct size significantly affects myocardial tissue and keeps on damaging with every passing second of ischemic time [17–19]. Hence, even with optimal reperfusion (primary PCI), prolonged ischemic time may cause higher mortality and less myocardial salvage [8, 9]. Decrease in DTB time is unlikely to render the ultimate desired reduction in mortality after primary PCI [13], and therefore, it should not be considered the sole quality indicator [15]. Therefore, the matrix of focus has to be shifted, along with strategies in place to reduce DTB time, TIT also need to bring to light in order to improve the survival from STEMI.

## Limitation

Single center registry-based study and non-randomized nature of the design are key limitations of this study. Secondly, time of onset of chest pain was noted in the ER based on patient or attendants recall, which may have some over or understating. Even though TIT was observed to be an independent predictor of in-hospital mortality, but low predictive value of ischemic time warranted further investigation for the potential effect of confounding factors.

## Conclusions

Prolonged TIT was associated with higher in-hospital mortality of STEMI patients in whom the recommended DTB was achieved. Although predictive value of TIT was low, but it was found to be an independent predictor of mortality and risk of mortality increases nearly by 5% with every passing hour of TIT. This could be achieved through a rigorous Emergency Management System (EMS) and meticulous patient awareness programs in developing countries.

## Data Availability

Data and material will be available upon request.

## Abbreviations

STEMI: ST elevation myocardial infarction
DTB: Door to balloon
TIT: Total ischemic time
PCI: Percutaneous coronary intervention
ER: Emergency room
EMS: Emergency Management System
